# Prevalence of Ocular Allergy Among High School Children in Trinidad and Tobago: A Cross‐Sectional Study

**DOI:** 10.1002/hsr2.70709

**Published:** 2025-05-04

**Authors:** Ngozika E. Ezinne, Narissa Gartiesingh, Ryan Poonan, Khathutshelo P. Mashige

**Affiliations:** ^1^ Optometry Unit, Department of Clinical Surgical Sciences University of the West Indies, Saint Augustine Campus Trinidad and Tobago; ^2^ Discipline of Optometry University of KwaZulu‐Natal Durban South Africa

**Keywords:** allergic conjunctivitis, atopy, high school children, prevalence, Trinidad and Tobago

## Abstract

**Background and Aims:**

Ocular allergy (OA) constitutes a significant global public health concern, affecting an estimated 15% to 20% of the world's population, with approximately 40% of cases occurring in children. In Trinidad and Tobago (T&T), the risk of OA is notably heightened due to its geographical location and the recurrent exposure to Sahara dust. This study seeks to determine the prevalence of ocular allergy among secondary school students in T&T.

**Method:**

A descriptive cross‐sectional study was conducted in a school‐based setting from October 2022 to May 2023. Schools were selected for inclusion using a randomization process facilitated by an online spinner wheel tool. Data were collected using a modified version of the International Study of Asthma and Allergies in Childhood (ISAAC) questionnaire. The questionnaire captured information on demographic characteristics, the presence of ocular allergy symptoms, associated risk factors, and the impact of ocular allergy. The data collected was analysed using the Statistical Package for Social Sciences (SPSS). The relationship between variables was assessed using Pearson Chi‐square test, with a *p*‐value of < 0.05 considered statistically significant.

**Results:**

A total of 420 students, aged 11–18 years, participated in the study, comprising 198 males (47.1%) and 222 females (52.9%). The overall prevalence of OA was 49.3% (*n *= 207), and seasonal allergic conjunctivitis (SAC) being the most common subtype, accounting for 90.3% of OA cases. The prevalence of OA was highest among 14‐year‐old age group (23.4%) and more frequent among females (58.8%). Factors significantly associated with OA included difficulty breathing or wheezing, asthma, food, rhinitis, atopic eczema, and exposure to mites (*p* < 0.05).

**Conclusion:**

The prevalence of ocular allergy (OA) among secondary school students in T&T was relatively higher compared to findings from various studies conducted globally. This underscores the need for implementing effective strategies for early diagnosis and management to mitigate its impact.

## 1 Introduction

The eye is a frequent site for the development of allergic inflammatory disorders due to the absence of a mechanical barrier to prevent the deposition of allergens, such as pollen, on its surface [1]. Ocular allergy (OA) is a group of hypersensitivity reactions to typically harmless substances, known as allergens, and may present as the primary manifestation of allergic sensitization [2]. When the immune system mistakenly identifies a benign substance as an allergen, it triggers an overreaction, producing antibodies that activate cells to release chemicals, resulting in an allergic response. This reaction manifests symptoms such as watery eyes, pruritus, redness, a gritty sensation, eyelid oedema, potential pain, and photophobia.

Ocular allergies encompass several subtypes, including seasonal allergic conjunctivitis (SAC) and perennial allergic conjunctivitis (PAC), vernal keratoconjunctivitis (VKC), atopic keratoconjunctivitis (AKC), and giant papillary conjunctivitis (GPC) [1, 3, 4].

Ocular allergy (OA) is triggered by various environmental, chemical, and systemic factors globally. Key triggers include exposure to allergens such as pollen, molds, dust, pets or animal fur, and smoke from wood or coal used for cooking. Indoor tobacco smoking, household heating, preservatives, fragrances, and dyes present in cosmetic products also contribute to OA [5, 6, 7]. Additionally, exposure metals like nickel, cobalt, chromium, and lead, found in concentrations exceeding the recommended limit of 1 ppm in products such as eye shadows, eyeliners, and toy makeup kits, were identified as a potential cause of OA [8]. Recent findings suggest that platinum salts, commonly used in jewellery, electrical, and automotive products, may also induce allergic reactions [3]. Co‐morbidities associated with OA include asthma, allergic rhinitis (AR), and atopic dermatitis [9]. If left undiagnosed or untreated, OA may lead to complications such as severe dry eye syndrome, meibomian gland dysfunction, and in some cases, keratoconus [10, 11, 12].

Allergic conditions are prevalent across all pediatric age groups, with an estimated prevalence of up to 40% among children [13]. While the symptoms of OA are not life‐threatening, they significantly impact productivity and quality of life. Children with OA often experience behavioural, emotional, and mental challenges as a result of their condition [14].

The management of OA poses a substantial challenge for both patients and eye‐care practitioners due to its chronic nature and limited public awareness [15]. Furthermore, OA is among the most commonly reported ocular conditions during out‐patient eye consultations, particularly in hot or tropical climates [15].

Numerous studies [4, 6, 15, 16] have investigated OA across diverse geographic regions. Evidence from research conducted in the United States, Sweden, Brazil, and Uganda indicated that ocular allergies affected approximately 15%–20% of the global population, corresponding to an estimated 1.58 billion individuals [15].

The prevalence of OA is influenced by demographic factors and environmental conditions, with children being more frequently affected than adults [16, 17].

The geographic location of T&T predisposes its population to an increased risk of ocular allergy, partly due to environmental factors such as Saharan dust from Africa. While numerous studies have investigated ocular allergy in adults, limited research has focused on children, and no studies to date have been conducted within the Caribbean, particularly in T&T. This underscores the need to evaluate the prevalence of OA in T&T to facilitate comparisons with findings from other regions. Consequently, this study aimed to determine the prevalence of OA among children in T&T.

## 2 Methods

**Figure 1:**
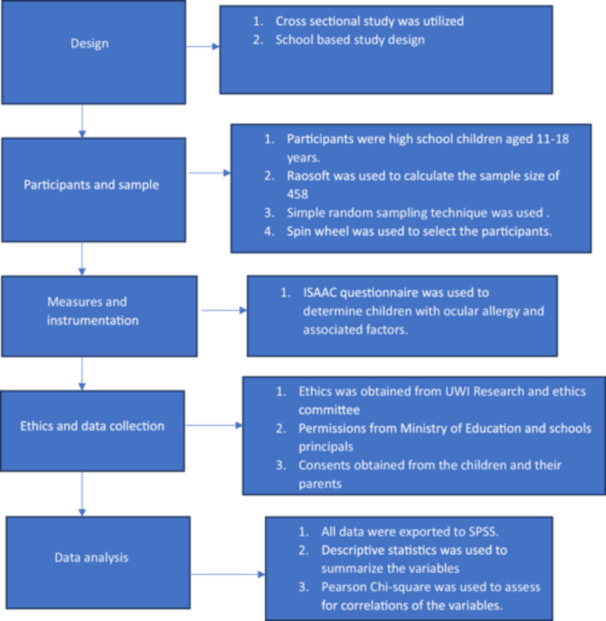
Study methodology.

The study methodology is illustrated in Figure 1.

### 2.1 Research Design and Setting

This study employed an institutional descriptive cross‐sectional survey design, targeting all high school students across selected high schools in distinct regions in T&T, namely Couva East Secondary, Arranguez North Secondary, and Barrackpore West Secondary School. The data collection was conducted between October 2022 and April 2023. T&T is an archipelagic state situated in the southern Caribbean, northeast of Venezuela and south of Grenada in the Lesser Antilles [18]. Trinidad, one of the twin islands of T&T, currently has a population of approximately 1.4 million people [19].

Trinidad is geographically divided into three main regions: northern, central, and southern areas. Each area encompasses the three primary categories of schools in T&T: co‐educational schools, girls‐only schools, and boys‐only schools. Within these categories, schools can be classified as fully government‐funded, partially government‐funded and private funded [20]. This study exclusively included fully government‐funded co‐educational schools, which accommodate both male and female students, to minimize bias and ensure a broader range of participants.

### 2.2 Inclusion and Exclusion Criteria

All students enrolled during the 2022/2023 academic year in the selected group of schools, aged between 11 and 18 years, were eligible to participate in the study. However, students presenting with symptoms of OA not attributable to external factors, such as previous surgical interventions, were excluded from participation.

### 2.3 Study Sample

The sample size was determined using the RAOSOFT sample size calculator. Based on a total population of 1,403,375 in T&T and a target population of 56,262 students enrolled in co‐educational schools, the calculator was configured with a 95% confidence interval and a 5% margin of error. This yielded a required sample size of 383 students. To account for potential nonresponse due to absenteeism or unwillingness to participate, the sample size was increased by 20%, resulting in an adjusted sample size of approximately 458 students. Therefore, the final total sample size was determined to be 458 participants.

### 2.4 Sampling Technique

A simple random sampling method was employed for participant selection. A comprehensive list of all the co‐educational fully government‐funded secondary schools in Trinidad was compiled and categorized according to geographic regions. Schools within each region were entered into a SpinWheelMaker. com platform, which was used to randomly automatically select one school from per region for region for inclusion in the study. The selected schools were Couva East Secondary, Barrackpore West Secondary, and Aranguez North Secondary, representing three regions in T&T. Schools within each region were entered into the SpinWheelMaker. com platform, which was used to randomly select one school per region for inclusion in the study. The selected schools were Couva East Secondary, Barrackpore West Secondary, and Aranguez North Secondary, representing three distinct regions in Trinidad.

The total sample size of 482 students was evenly disturbed among the three selected schools, resulting in approximately 160 students per school. To allocate students across grade levels, the number of students per school (160) was divided by five, as most secondary schools in T&T have five, grade levels, (Forms 1 to 5). A minimum of 30 students were recruited from each level.

### 2.5 Data Collection Tool

The data were collected using the International Study of Asthma and Allergies in Childhood (ISAAC) questionnaire, a standardized instrument widely applied in epidemiological research [2, 7, 16, 17]. This tool is designed to assess the prevalence and severity of asthma and allergic conditions, including allergic rhinitis and eczema, among children and adolescents. In this study, the questionnaire included specific items related to OA, alongside questions addressing demographic and patient‐related data. The current study utilized a modified version of the questionnaire previously employed in a similar study conducted in China [16]. A pilot study involving 20 children outside the study area was conducted to assess the questionnaire's applicability. Based on the feedback obtained during this pilot study, adjustments were made to refine the final version of the data collection instrument used in the main study.

### 2.6 Data Collection

Ethical approval for the study was obtained from the Research and Ethics Committee of the University of the West Indies (UWI). Additionally, authorization to conduct the research within the selected co‐educational government schools was granted by the Ministry of Education and the principals of the respective schools. N.G. and R.P. conducted visits to the schools, during which meetings with the students were arranged. The purpose and nature of the study were thoroughly explained to the students during the meetings. A list of students, provided in various formats by the classroom teachers, was entered into Spinner Wheel application. From this, 30 students were randomly selected and subsequently invited to participate in the study. Participation was contingent on obtaining informed consent from the selected students. Upon receiving consent, data collection was through face‐to‐face distribution of ISAAC questionnaires. The students were given a 1‐month period to complete and return the questionnaires, with assistance provided to those who encountered difficulties in completing them.

### 2.7 Data Analysis

All collected data were entered into Microsoft Excel and subsequently exported to Google Sheets for data organization. The finalized data set was then imported into the Statistical Package for Social Sciences (SPSS), version 27, for analysis. Descriptive statistics were performed, and prevalence rates were calculated as percentages for various variables. Associations between variables were assessed using Pearson's chi‐squared tests, with statistical significance set at a *p*‐value of < 0.05.

### 2.8 Ethical Consideration

Ethical approval for the study was obtained from the Research and Ethics Committee of the University of West Indies (CREC‐SA/1799/10/2022). Permission to conduct the study in high schools was granted by the Ministry of Education and the school principals. Written informed consent was obtained from the parents of participating students, and assent was secured from all students where applicable. The study was conducted in accordance with the principles outlined in the Declaration of Helsinki. No personal identifiers were collected, and strict confidentiality was maintained for all data obtained during the study.

### 2.9 Criteria for Diagnosis

A positive symptom of OA was defined as a self‐reported history of itchy/watery eyes, with at least one episode experienced in the past 12 months. Seasonal OA or Seasonal Allergic Conjunctivitis (SAC) was characterized by symptoms occurring over two or more consecutive months. Perennial OA or Perennial allergic conjunctivitis (PAC) was classified by symptoms persisting for four or more months within the same year.

## 3 Results

### 3.1 Demographic Profile of Study Participants

A total of 420 participants, aged 11 to 18 years (mean ± SD: 14.36 ± 22.65 years) were included in the study, yielding a response rate of 87.1%. The majority (*n* = 320, 76.2%) resided in tree‐dense areas, and approximately half (*n* = 222, 52.9%) were females (Table 1).

**Table 1:** Demographic profile of participants.

Variables	Subgroup	Frequency (*N* = 420)
Gender	Male	198 (47.1)
	Female	222 (52.9)
Age (years)		
	≤ 12	54 (12.9)
	13	72 (17.1)
	14	108 (25.7)
	15	78 (18.6)
	16	74 (17.6)
	≥ 17	34 (8.1)
Form		
	Form 1	100 (23.9)
	Form 2	75 (17.9)
	Form 3	112 (26.8)
	Form 4	53 (12.7)
	Form 5	78 (18.7)
Lives around a lot of trees/grass		
	Yes	320 (76.2)
	No	100 (23.8)

### 3.2 Prevalence of OA Among the Participants

**Figure 2:**
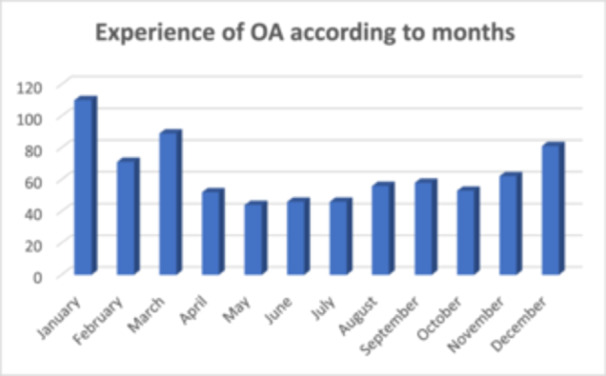
Experience of OA according to months.

The prevalence of OA in this study was 49.3% (*n* = 207). Seasonal allergic conjunctivitis (SAC) was the most common subtype, accounting for 90.3% (*n* = 197) of cases. Most participants reported experiencing OA symptoms in January (*n* = 110, 26.3%) (Figure 2). A statistically significant association was observed between the month and the occurrence of symptoms (*p* < 0.001) (Table 2).

**Table 2:** Prevalence of OA.

Variables	Subgroup	Frequency (%) (*N* = 420)
Presence of OA	Yes	207 (49.3)
	No	212 (50.5)
	I cannot remember	1 (0.2)
Gender distribution of OA (*n* = 207)		
	Male	85 (41.5)
	Female	122 (58.8)
Age distribution of OA (*n* = 207)		
	≤ 12	29 (14)
	13	32 (15.4)
	14	48 (23.4)
	15	32 (15.4)
	16	44 (21.2)
	≥ 17	22 (10.6)
Distribution of type of OA (*n* = 207)		
	Perennial ocular allergy	20 (9.7)
	Seasonal ocular allergy	187 (90.3)
Associated symptoms experienced		
	Redness	207
	Photophobia	73 (26.4)
	Foreign body sensation	74 (26.5)
Severity of symptoms		
	I cannot remember	89 (29.2)
	1–4 times (mild)	27 (6.4)
	5–10 times (moderate)	37 (8.8)
	More than 10 times (severe)	55 (13.1)

### 3.3 Concurrence of OA With Other Allergic Conditions

The most prevalent allergic condition related to OA was difficulty breathing or wheezing (27.2%), followed by asthma (9.5%) and food allergies (8.3%) (Table 3). OA demonstrated a significant association with breathing difficulties and wheezing, allergic rhinitis, atopic eczema, and pollen allergy (All *p* < 0.05).

**Table 3:** Concurrence of OA with other allergic conditions (*n* = 207).

Variable	Frequency (%)	*p* value
Had trouble breathing and wheezing sound in the last 12 months?		
Yes	114 (55.1)	
No	93 (44.9)	< 0.001
Diagnosed with allergic rhinitis?		
Yes	23 (11.1)	
No	184 (88.9)	0.016
Diagnosed with asthma?		
Yes	40 (19.3)	0.216
No	167 (80.7)	
Diagnosed with atopic eczema?		
Yes	18 (8.7)	0.020
No	189 (91.3)	
Do you have a pollen allergy?		
Yes	29 (14.0)	0.013
No	178 (86.0)	
Do you have any food allergies?		
Yes	35 (16.9)	0.140
No	172 (83.1)	
Do you have a mite allergy?		
Yes	11 (5.3)	0.198
No	196 (94,7)	
Do you wear contact lenses?		
Yes	2 (1.0)	0.548
No	205 (99.0)	

### 3.4 Impact of OA on Daily Activities

**Figure 3:**
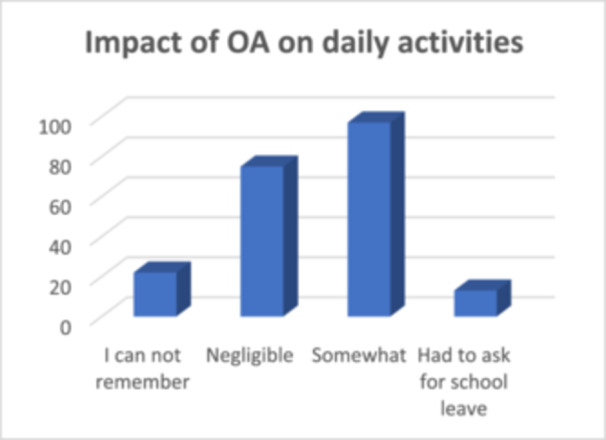
Impact of OA on daily activities.

OA had minimal impact on the daily activities of the study participants, as only a small proportion sought medical consultation (*n* = 26, 12.6%) or required leave of absence from school (*n* = 13, 6.3%) (Figure 3). Furthermore, the impact of OA was significantly associated with the month of OA occurrence (*p* < 0.001), difficulty in breathing (*p* < 0.001), challenges with food consumption (*p* < 0.05), pollen allergy (*p* < 0.01) and sensitivity to mites (*p* < 0.04).

## 4 Discussion

To the best of our knowledge, this study is the first to evaluate the prevalence of OA among high school students in the Caribbean, specifically in T&T. The findings indicate that OA is a significant condition within this population, with the highest prevalence observed among students aged 14 years or younger. Additionally, the study identified SAC as the most common subtype of OA. Breathing difficulties or wheezing were most frequently associated allergic conditions reported alongside OA. The prevalence of OA recorded observed in this study was higher than the rates of 39.9%, 32%, 19.2%, and 17.5% reported in studies conducted among school children in other countries with comparable climatic conditions, such as Ghana [21], Nigeria [15], Pakistan [22]. Additionally, it exceeded the prevalence reported in Japan [23]. It might be expected that the prevalence would align more closely with countries with similar weather conditions, such as Ghana or Nigeria. However, genetic differences and variations in the assessment methodologies for ocular symptoms could explain the discrepancies. For instance, the omission of anterior eye assessments for OA signs in other studies could account for the high prevalence of OA observed in the study. In the present study, redness was identified as the most prevalent symptom of OA, observed in 100% of cases. Similar findings were reported in studies conducted in Shanghai China [16], Hyderabad India [24], and Brazil [13]. However, other studies highlighted photophobia and sticky discharge as the most commonly associated OA symptoms [15, 25]. The predominance of redness in this study is consistent with its frequent occurrence in children exposed to environmental allergens. The conjunctiva, with its dense network vasculature, is particularly susceptible to allergen‐induced inflammation. This inflammatory response leads to vasodilation, which clinically manifests as the characteristic redness observed in allergic conjunctivitis.

Our study demonstrated a higher prevalence of OA among females compared to males. This gender disparity may be attributed to hormonal influences, differences in immune system function, and behavioural factors. Similar trends were reported in epidemiological studies conducted in North Central Nigeria [15], Gambia [25] and Uganda [26]. Conversely, cross‐sectional surveys conducted in Chandigarh, India, China and Pakistan documented a higher prevalence of OA among males [14, 17, 22]. These discrepancies may stem from variations in immune system characteristics and other contextual factors. In our study, the prevalence of OA was higher among 14‐year‐olds, with symptoms predominantly observed in individuals aged 14 to 16 years. Comparable findings were reported by Kumah et al. [21] in a study conducted in the Kumasi Metropolis, which identified the highest prevalence of OA among adolescents aged 13–16 years. This increased prevalence among adolescents aged 14 years and older is likely attributable to a combination of factors, including enhanced environmental exposure, immunological development, and behavioural patterns. The higher incidence of allergic conjunctivitis adolescents compared to younger children can be attributed to several factors. Adolescents are typically exposed to a broader spectrum of environmental allergens due to increased outdoor activities and participation in sports, which enhance contact with potential allergens such as pollen, dust, and mould. Moreover, by the age of 14, the immune system undergoes significant maturation, leading to a more pronounced immune response that may exacerbate allergic symptoms. Furthermore, the process of allergen sensitization often intensifies during puberty and adolescence, contributing to the increased prevalence of allergic conjunctivitis within this demographic.

Contrary to previous reports suggesting that PAC is common in tropical climates [4], our study identified SAC as the most prevalent type of OA in T&T, despite its tropical island setting. These findings underscore the necessity for further population‐based studies on OA within Trinidad to corroborate and contextualize our results. The elevated prevalence of OA symptoms observed in January may be attributed to the seasonal Sahara dust surge, typically occurring between December and January in T&T [27]. Furthermore, the increase in symptoms in January could be attributed to the dry season in T&T, a period characterized by elevated levels of airborne dust and pollen. In contrast to our findings, a study conducted in Nigeria by Malu [15] reported a higher prevalence of OA symptoms in July. Given that both T&T and Nigeria share tropical climates, similar prevalence patterns of ocular allergies might be anticipated. Additional research is necessary to explore and elucidate the factors underlying these variations in findings.

Atopic diseases, including allergic rhinitis, pollen allergy, respiratory difficulty, and wheezing, were identified as major co‐occurring allergic conditions associated with OA in the present study. These findings align with previous research by Feng et al. [16], Geraldini et al. [13], and Pitchon et al. [28], which suggests that OA frequently coexists with at least one allergic condition. In contrast, studies conducted in Nigeria [15] and The Gambia [25] reported refractive errors as the primary coexisting condition with OA. Additionally, other ocular conditions, such as pinguecula, pterygium, corneal ulcers, abrasions, scars, and keratoconus, were also documented to be associated with OA [25].

The majority of our study participants (87.4%) reported that their daily activities were not significantly affected by OA. This finding aligns with expectations, as SAC typically exerts a minimal impact compared to PAC, which is more strongly associated with rhinitis and generally linked to greater interference with daily activities. Similar findings were reported in studies in Ghana [21]. However, OA was reported to result in economic losses, missed employment, and educational opportunities, ultimately contributing to a reduced quality of life in Europe [29]. Futhermore, studies from Europe recorded a significant economic burden of OA, with over 300,000 Euros expended annually on management, particularly in children, underscoring its substantial public health concern [29, 30, 31]. Additionally, OA though not reported to have impacted academic performance in the current study, was recorded to have negative impacts on academic performance, physical activity, sleep, and emotional well‐being [32]. Therefore, implementing strategies for early detection and effective management of OA is critically important.

### 4.1 Strengths and Limitations of the Study

Our study has several notable limitations. Data collection was conducted through self‐reported questionnaires, which may be subject to recall bias. Additionally, unlike other studies, we did not assess the anterior segment for OA signs, limiting our ability to identify cases of vernal keratoconjunctivitis (VKC). The small sample size and the inclusion of only high school students further constrain the generalizability of our findings, as younger children were excluded. Furthermore, as a cross‐sectional study, we were unable to establish causal relationships or temporal associations between the observed variables. Lastly, the school‐based nature of the study restricts its population‐based applicability, and a longitudinal design would have provided more comprehensive insights into the profile and progression of allergic conditions among students in T&T.

Despite the limitations of our study, the sample size was sufficiently large to provide valuable insights into the prevalence of OA in the Caribbean, particularly in T&T. Furthermore, the study employed the ISAAC questionnaire, a well‐established tool that utilizes standardized methods for assessing OA symptoms. This enables robust comparisons across different countries and facilitates the analysis of data collected at various time points.

## 5 Conclusion

The prevalence of ocular allergy (OA) among high school students in T&T is notably high, representing a significant public health concern. Consequently, it is imperative to implement targeted strategies aimed at raising awareness, enhancing early detection, and improving the management and prevention of OA in this cohort.

## Author Contributions

**Ngozika E. Ezinne:** conceptualization, methodology, supervision, writing – original draft, writing – review and editing, validation. **Narissa Gartiesingh:** investigation, project administration, resources, data curation, formal analysis, visualization, writing – original draft, methodology. **Ryan Poonan:** investigation, methodology, formal analysis, data curation, resources, project administration, visualization, writing – original draft. **Khathutshelo P. Mashige:** supervision, writing – review and editing, validation, methodology.

## Conflicts of Interest

The authors declare no conflict of interest.

## Transparency Statement

The lead author Ngozika E. Ezinne affirms that this manuscript is an honest, accurate, and transparent account of the study being reported; that no important aspects of the study have been omitted; and that any discrepancies from the study as planned (and, if relevant, registered) have been explained.

## Data Availability

The data that support the findings of this study are available from the corresponding author upon reasonable request. All authors have reviewed and approved the final version of the manuscript. The corresponding author retains full access to all data associated with this study and assumes full responsibility for the integrity of the data and the accuracy of the data analysis.
